# Hyperfibrinolysis Detection During Liver Transplantation Using Viscoelastometry

**DOI:** 10.1111/ctr.70179

**Published:** 2025-05-11

**Authors:** Sarah Thaler, Anna Zorn, Isabell Aster, Dionysios Koliogiannis, Bernhard W. Renz, Markus Guba, Philipp Groene

**Affiliations:** ^1^ Department of Anaesthesiology LMU University Hospital, LMU Munich Munich Germany; ^2^ Department of General‐, Visceral‐ and Transplant‐Surgery LMU University Hospital, LMU Munich Munich Germany

**Keywords:** coagulation, ECA‐test, hyperfibrinolysis, liver transplantation, viscoelastometry

## Abstract

**Background:**

End‐stage liver disease induces a precarious hemostatic equilibrium, named rebalanced hemostasis. Liver transplantation additionally causes profound disturbances in the hemostatic balance. Hyperfibrinolysis poses a relevant impairment to the coagulation process during liver transplantation. During surgery, the hemostatic management is guided by viscoelastic monitoring systems. The aim of this prospective, observational study was to evaluate the incidence of hyperfibrinolysis during liver transplantation using different viscoelastic assays, namely an ecarin‐based test and a tissue factor‐based test.

**Methods:**

Blood sampling was done at five measurement time points during liver transplantation (T1 induction of general anesthesia, T2 start of anhepatic phase, T3 end of anhepatic phase, T4 10 min after reperfusion, T5 end of surgery). Viscoelastic testing included ClotPro assays EX‐test, FIB‐test, AP‐test, and ECA‐test. Hyperfibrinolysis was defined as a maximum lysis of at least 15%. Lysis detection time (LDT) served as an indicator for the velocity of lysis, marking the time point when less than 85% of the clot are extant.

**Results:**

Thirty transplantation surgeries were included. A total of 150 viscoelastic measurements have been performed. The ECA‐test detected hyperfibrinolysis significantly more often (31 [21%] vs. 22 [15%] out of 150, *p* = 0.039) and in a higher number of patients than the EX‐test. The ECA‐test revealed hyperfibrinolysis significantly earlier compared to the EX‐test (median LDT 2100 s [1500/2900] vs. 3300 s [2400/3800], *p* < 0.001).

**Conclusion:**

This study demonstrates higher sensitivity of the ecarin‐test than the tissue‐factor‐test in monitoring hyperfibrinolysis, with more frequent and earlier detection of this coagulopathy.

**Trial Registration:**

German Clinical Trials Register: DRKS00032827

## 1 Introduction

The balance between blood clot formation and breakdown relies significantly on liver function, as the liver is responsible for producing and breaking down numerous pro‐ and anticoagulant factors [1]. Liver cirrhosis and end‐stage liver disease induce a precarious hemostatic equilibrium, referred to as rebalanced hemostasis, that may result in bleeding or thrombosis. Both of these conditions may exist simultaneously [2, 3, 4].

For end‐stage liver disease, orthotopic liver transplantation is the only definitive treatment. In addition to liver dysfunction‐related hemostatic impairment, liver transplantation causes significant disruptions in the hemostatic balance due to intraoperative blood loss and varying liver function during the distinct phases of surgery [3, 4]. End‐stage liver disease may lead to a reduced amount of fibrinogen as well as to structural modifications of the protein itself. In either case, it implicates altered functionality and clot lysis, which is termed hyperfibrinolysis [4, 5, 6]. This condition, being manifested by a premature clot depletion, poses a relevant impairment to the coagulation process during liver transplantation, potentially increasing the risk of bleeding. Through the accumulation of tissue plasminogen activator (t‐PA) due to reduced or missing hepatic clearance during the anhepatic phase and after reperfusion, progression towards hyperfibrinolysis and bleeding risk gets aggravated [7, 8]. As hyperfibrinolysis can effectively be addressed by antifibrinolytic drugs, such as tranexamic acid, its early and precise detection has direct clinical significance. Managing coagulation during liver transplantation is highly challenging. Coagulation monitoring, therefore, needs to ensure early detection of pathological conditions and timely intervention.

Conventional laboratory coagulation tests are of limited predictive value for coagulation‐related bleeding complications and transfusion requirements [2, 3, 9]. Measuring single endpoints, these tests do not appropriately reflect the complexity of the coagulation system. Furthermore, due to the measurement procedure they can only provide a delayed result so that physicians have to react to a potentially outdated finding. Notably, certain pathological conditions, such as hyperfibrinolysis, are not diagnosed by these tests at all.

In liver transplantation, viscoelastic monitoring systems like ROTEM (Werfen, Barcelona, Spain) or ClotPro (Haemonetcis, Boston, United States of America) provide earlier, more comprehensive, and patient‐specific guidance for hemostatic management. Analyzing the kinetics of coagulation from clot initiation over clot strength to clot degradation viscoelastometry enables a functional assessment of the entire coagulation process [10, 11, 12]. Both systems provide comparable standard assays as well as comparable variables. The ClotPro system additionally provides test assays for direct oral anticoagulant detection and evaluation of fibrinolysis. The ROTEM system is available both as a cartridge‐based system and a pipet‐based system. The ClotPro system is a pipet‐based system with active tip technology reducing reagent handling.

The viscoelastic standard assays allow for the evaluation of the extrinsic and intrinsic pathway as well as the fibrinogen status. Hyperfibrinolysis is also revealed in these assays. ClotPro's ecarin‐based ECA‐test derived from the snake venom Ecarin appears to detect hyperfibrinolysis earlier and more frequently than viscoelastic standard assays [13].

The aim of this prospective observational study was to evaluate the incidence of hyperfibrinolysis during liver transplantation using two different viscoelastic assays, namely the ecarin‐based ECA‐test and the tissue factor‐based EX‐test. Additionally, a comparative evaluation of the lysis detection time (LDT) was performed. The hypothesis tested was that the ECA‐test allows earlier and more frequent detection of hyperfibrinolysis in vivo than the EX‐test.

## 2 Methods

The study protocol was approved by the Ethics Committee of Ludwig Maximilians University (LMU), Munich, Germany (No 23‐0613). The study was conducted in accordance with the Declaration of Helsinki. Written informed consent was obtained from all patients prior to study inclusion. Exclusion criteria comprised age <18 years and refusal to participate in the study. The study was registered at the German Clinical Trials Register (DRKS00032827) before inclusion of the first patient.

Blood sampling was done at five measurement time points (T1 induction of general anesthesia, T2 start of anhepatic phase, T3 end of anhepatic phase, T4 10 min after reperfusion, T5 end of surgery). Viscoelastic testing was performed at each of the measurement time points using the ClotPro analyzer (Haemonetics, Boston, United States).

Viscoelastic testing included the assays EX‐test, FIB‐test, AP‐test as well as ECA‐test. EX‐test allows for the evaluation of the extrinsic pathway initiating clot formation by addition of tissue factor. FIB‐test specifically assesses the fibrinogen status. AP‐test enables the confirmation of hyperfibrinolysis using aprotinin as fibrinolysis inhibitor. ECA‐test deploys the snake venom ecarin from the viper species *Echis carinatus*. Ecarin engages the common pathway and activates prothrombin to meizothrombin followed by the conversion of fibrinogen to fibrin. Notably, the ECA‐test does not contain calcium, whereas all the other assays contain calcium chloride. All tests were performed under standardized conditions at 37 degrees and ran for 75 min each. The following viscoelastic variables were documented: clotting time (CT), clot formation time (CFT), A5 and A10 (clot amplitude 5 and 10 min after CT), maximum clot firmness (MCF), and maximum lysis (ML). According to the manufacturer's instructions, hyperfibrinolysis was defined as ML of at least 15%, that is, a reduction of the MCF of at least 15% at any time during the testing period. Additionally, the LDT was recorded, marking the time point when less than 85% of the clot are extant. This variable serves as an indicator for the velocity of lysis. At measurement time points T1, T3, and T5 the conventional laboratory coagulation values activated prothrombin time (aPTT) and internationalized normalized ratio (INR) were determined additionally.

### 2.1 Statistics

Based on data from Zatroch et al. [13] and Abuelkasem et al. [14] regarding the occurrence of hyperfibrinolysis during liver transplantation using viscoelastometry with an expected detection rate of 36% to 46% (5%–10% absolute difference between the different viscoelastic tests) 30 pairs (×5 measurements = 150 samples) were to be examined to achieve a power of 80% (McNemar test). A non‐parametric Friedman's test was used for the comparison of different results over time. Data are presented as median with interquartile range (Q1/Q3) unless otherwise indicated. Statistical analysis was performed using GraphPad Prism 10 (La Jolla, USA). An alpha error <0.05 is considered statistically significant.

## 3 Results

Thirty‐one liver transplant procedures at LMU University Hospital between September 2023 and June 2024 were included in the study. One patient was dropped out due to missing data. Three of the patients were recorded twice in the dataset due to re‐transplantation. A total of 150 measurements have been performed. Patient characteristics and the etiology of liver dysfunction, as well as surgical data on the length of surgery and the distinct surgery phases, blood loss, transfusion volumes, and coagulation substitutes, are presented in Table 1.

**TABLE 1:** Demographics of patients and surgery data.

Age (years)	55 (45/60)
Sex (male/female)	20 (67%) / 10 (33%)
BMI (kg/m^2^)	28 (21/31)
Etiology of liver pathology	
Alcoholic liver disease	8 (27%)
Biliary cirrhosis	8 (27%)
Autoimmune hepatitis	3 (10%)
Re‐Tx due to graft failure	3 (10%)
HCC	2 (7%)
M. Wilson	1 (3%)
Others	5 (17%)
Child‐Pugh‐Score (*n* = 27)	
Child A	2 (7%)
Child B	10 (33%)
Child C	15 (50%)
MELD‐Score	23 (15/30)
Length of surgery (min)	291 (243/359)
Preparation phase (min)	86 (70/107)
Anhepatic phase (min)	63 (46/97)
Neohepatic phase (min)	129 (107/174)
Blood loss (mL)	3500 (1900/6850)
Red Blood Cell concentrate (mL)	1000 (500/1563)
Autologous blood transfusion (mL)	648 (280/1327)
Platelet concentrate (mL)	300 (0/600)
Fresh Frozen Plasma (mL)	1875 (1313/3000)
Fibrinogen (g)	4.5 (1.5/8)
Prothrombin complex concentrate (I.E.)	1000 (0/3150)
Tranexamic acid	10 (33%)

The fibrinolytic activity might rise at any phase throughout liver transplantation so that hyperfibrinolysis partially was detected in a patient at multiple measurement time points. The application of tranexamic acid was at the anesthesiologist's discretion independent of the study measurements. It was based on the clinical presentation of bleeding and intraoperatively conducted ROTEM measurements, which is the in‐house standard of viscoelastometric monitoring. Ten out of 30 patients received tranexamic acid intraoperatively.

In the EX‐test, hyperfibrinolysis was detected in 37% of the patients (11 out of 30 patients). In the ECA‐test, hyperfibrinolysis detection rate was higher with 53% of all patients (16 out of 30 patients). In one patient, hyperfibrinolysis was indicated in the EX‐test alone, in six patients it was indicated in the ECA‐test alone.

Divided into surgical phases, the peak of hyperfibrinolysis was found at the end of the anhepatic phase and shortly after reperfusion. The measurement time points T3 and T4 accounted for 23% and 41%, respectively, in the EX‐test and for 26% and 42%, respectively, in the ECA‐test.

Out of 150 measurements across all patients in the EX‐test hyperfibrinolysis was observed in 15% of samples (22 out of 150). In the ECA‐test, hyperfibrinolysis was detected in 21% of samples (31 out of 150), with the ECA‐test identifying a hyperfibrinolytic status significantly more frequently (*p* = 0.039). Both assays detected hyperfibrinolysis in 13% of all measurements (19 out of 150), simultaneously. This indicates that hyperfibrinolysis was observed exclusively in the EX‐test in three samples and exclusively in the ECA‐test in 12 samples.

The AP‐test reversed the fibrinolytic activity in 17 out of 19 measurements when hyperfibrinolysis was detected in both assays EX‐test and ECA‐test.

In all 12 measurements where only the ECA‐test indicated hyperfibrinolysis this condition was resolved in the AP‐test. Among the three cases with hyperfibrinolysis identified solely by the EX‐test, the AP‐test abolished it in one instance. In the remaining two cases, fibrinolytic activity slightly exceeded the hyperfibrinolysis threshold (15% ML) and remained unchanged in the AP‐test.

### 3.1 Lysis Detection Time

The median LDT in the EX‐test was 3300 s (2400/3800), while it was only 2100 s (1500/2900) in the ECA‐test (*p* < 0.001). Thus, the ECA‐test revealed hyperfibrinolysis significantly earlier compared to the EX‐test.

## 4 Discussion

The uncertainty about the mechanistic role of hyperfibrinolysis in bleeding complications in the context of liver‐related rebalanced hemostasis and liver transplantation is partially based on the lack of laboratory tests for its evaluation. Laboratory tests only cover individual components rather than providing a comprehensive overview of coagulation comprising both the pro‐ and antifibrinolytic portion. A key advantage of viscoelastic instruments is their ability to measure hyperfibrinolysis as a possible cause of bleeding [15].

In a comparison of various viscoelastic assays this study showed that the ECA‐test detects hyperfibrinolysis significantly more frequently and earlier than the EX‐test. This finding may be attributed to the unique properties of each assay. First, the ECA‐test utilizes ecarin, which directly activates the final pathway of coagulation by converting prothrombin to meizothrombin, which in turn cleaves fibrinogen to fibrin [16]. This bypasses most of the coagulation cascade, resulting in faster clot formation and faster detection of fibrinolytic activity, including hyperfibrinolysis. Second, unlike other assays, the ECA‐test does not contain calcium, thereby mitigating calcium‐dependent reactions in the hemostatic system including the activation of coagulation factor XIII (F XIII), the final stabilizer of fibrin, and thrombin‐activatable fibrinolysis inhibitor (TAFI) both of which exert antifibrinolytic effects [17, 18, 19, 20]. In chronic liver disease, the levels of F XIII and TAFI are already reduced, making clots in the ECA‐test sample more susceptible to fibrinolysis [21, 22, 23]. This characteristic may enable the ECA‐test to detect fibrinolytic tendencies more sensitively than other viscoelastic assays. This finding highlights the ability to detect hyperfibrinolyis is impacted by the viscoelastic testing methodology.

The EX‐test employs tissue factor to extrinsically activate the hemostatic cascade. Consistent with relevant literature this assay possesses higher sensitivity for the detection of hyperfibrinolysis compared to intrinsically activated assays [24]. However, the ECA‐test in this study allowed for more frequent and earlier detection of increased fibrinolytic activity emphasized by a significantly shorter LDT. This novel parameter recently introduced by Zatroch et al. for comparability between viscoelastic assays describes the period from the initiation of the coagulation process to an MCF decrease of at least 15% [13]. This way, the parameter accounts for the timing of hyperfibrinolysis detection, reflecting the velocity of the lysis process. The clinical relevance of the ECA‐test's higher hyperfibrinolysis detection rate depends on distinguishing whether the EX‐test lacks sensitivity to in vivo‐hyperfibrinolysis or whether the ECA‐test “hypersensitively” detects fibrinolysis that does not actually occur in vivo. The EX‐test likely aligns more closely with physiological conditions as it captures a broader range of the in vivo coagulation cascade, and contains calcium, an essential component of hemostasis in the body. The absence of calcium in the ECA‐test could potentially introduce an artificial “hypersensitivity” to fibrinolytic tendencies, which may not reflect in vivo hemostatic integrity. Therefore, the presence or absence of calcium might influence both sensitivity and specificity in detecting fibrinolytic activity. In vitro experiments adding calcium to the ECA‐test could contribute to elucidating the role of calcium in hyperfibrinolysis detection.

Viscoelastometry easily provides information about severe forms of hyperfibrinolysis, as indicated by a considerable breakdown of the clot in extrinsic and intrinsic assays. In contrast, monitoring of the normal fibrinolytic activity, which is an integral part of hemostasis, and subtle forms of hyperfibrinolysis remains challenging. In this study, the AP‐test served to verify hyperfibrinolysis. The AP‐test uses aprotinin to inhibit fibrinolysis. While in the other assays the clot is prematurely diminished or completely removed, prevention of clot degradation in the AP‐test is indicative for hyperfibrinolysis. In all cases of clot degradation in the ECA‐test this was abolished in the AP‐test confirming hyperfibrinolysis. The EX‐test showed the same result except for two cases when a minimal dissolution of the clot was present in the AP‐test and the EX‐test alike. Although hyperfibrinolysis cannot definitively be excluded as the cause of clot degradation, this rather argues for a reduced clot stability, for example, due to a lack of F XIII. This aspect, which was not part of the present study, should specifically be evaluated in future studies. Only the AP‐test served as a confirmatory test for hyperfibrinolysis, as there are no other validated tests for this coagulopathy.

Tranexamic acid, as an antifibrinolytic agent, holds prothrombotic properties. A shift in the hemostatic balance towards a prothrombotic state can increase the rate of postoperative thrombosis (e.g. portal vein thrombosis) and lead to catastrophic outcomes. Therefore, potent antifibrinolytic drugs like tranexamic acid should only be applied when hyperfibrinolysis is clinically suspected or detected. During liver transplantation hyperfibrinolysis mostly occurs intermittently and is often self‐limiting (e.g., after reperfusion) when the liver graft takes up its function [8, 25, 26]. In this study, the highest incidence of hyperfibrinolysis was observed in the anhepatic and immediate post‐reperfusion phase, which is in line with relevant literature [8, 25, 26]. Coagulation management intraoperatively requires repeated surveillance combined with a thorough clinical consideration of the necessity of tranexamic acid. In liver transplantation, instead of a preventive approach, mostly the therapeutic application of antifibrinolytic agents like tranexamic acid is pursued, if clinically justified, once hyperfibrinolysis has been diagnosed [27, 28].

The reason for the frequent measurements in the study setting was, on the one hand, to obtain a sufficient number of samples for comparison. On the other hand, the aim was to determine whether hyperfibrinolysis occurs more often than expected in the distinct surgical phases. The extent to which this requires treatment must be answered by further studies, especially regarding clinical events of bleeding, rather than merely focusing on measurement results.

Hypothermia is a frequent issue during liver transplantation and causes hemostatic impairments including an imbalance in the fibrinolytic system. While the precise mechanisms underlying temperature‐related hemostatic adjustments remain not fully understood, hypothermia has been linked with markedly diminished fibrinolysis induced by recombinant tissue‐type plasminogen activator (rt‐PA) as evidenced by a decrease in the Clot Lysis Index (CLI45) after 45 min in viscoelastic measurements [29]. Viscoelastic machines in clinical use standardly warm the blood sample to 37 degrees, which means they may not adequately reflect hypothermic coagulopathy and could fail to represent the in vivo hemostatic state during hypothermic phases of liver transplantation. Warming of the blood sample might artificially exaggerate fibrinolysis levels, which may not actually be as pronounced in the patient, potentially leading to unnecessary treatment of a presumed hyperfibrinolysis and an increased risk of thrombosis. Conversely, true in vivo‐hyperfibrinolysis might be masked by hypothermia, only to manifest upon rewarming of the body, which could then promote the occurrence of hyperfibrinolysis along with a risk of bleeding. A detailed analysis of pro‐ and anticoagulant proteins regarding quantity, activity, and structural modifications could help understand hypothermia‐related alterations of the fibrinolytic process.

Beyond liver pathologies and liver transplantation, early detection of hyperfibrinolysis, allowing for timely intervention, is also crucial in other scenarios prone to this coagulopathy such as polytrauma, postpartum hemorrhage, and pancreatic or cardiovascular surgery [30, 31, 32]. Thus, the ECA‐test might prove advantageous for a broader spectrum of disorders enabling a more targeted use of tranexamic acid and avoiding unnecessary application.

This study holds limitations, most importantly, the absence of a non‐viscoelastic standard method to confirm hyperfibrinolysis. Nevertheless, the extrinsic viscoelastic tests are the current standard for the detection of hyperfibrinolysis. By investigating only ClotPro assays, this study does not provide a clinical comparison to other point‐of‐care modalities that should be part of future studies. This study only focused on a selected group of patients and left other clinical patterns at risk of overactive fibrinolysis disregarded. The special group of liver transplant patients included in the study may limit the generalizability of the findings. Future studies will need to address other cohorts such as polytrauma patients and additionally shed light on the clinical impact in managing hyperfibrinolysis using this method. Last, in liver transplantation, the absence of the hepatic function, at least in part, translates to reduced levels of calcium in the body. The correlation of viscoelastically detected hyperfibrinolysis with intraoperative calcium levels may hold further diagnostic potential.

In summary, this study demonstrates the ecarin‐based‐test's higher sensitivity compared to the tissue‐factor‐based test for monitoring hyperfibrinolysis, as it enables more frequent and earlier detection of this coagulopathy. These findings may provide a rationale to earlier and more targeted antifibrinolytic therapy if clinically indicated, helping to avoid unnecessary application. This particularly applies to patients at simultaneous risk of bleeding and thrombosis in a precarious state of rebalanced hemostasis.

## Author Contributions

Sarah Thaler and Philipp Groene: initiated the study, conceptualized the study, created the methodology, handled data curation and software, and visualized the study. Anna Zorn: conducted the investigation. Sarah Thaler, Anna Zorn, Isabell Aster, and Philipp Groene: conducted formal analysis and data interpretation. Sarah Thaler, Isabell Aster, and Philipp Groene: wrote the original draft. Philipp Groene: provided supervision and project administration. Sarah Thaler, Anna Zorn, Isabell Aster, Dionysios Koliogiannis, Bernhard W. Renz, Markus Guba, and Philipp Groene edited the draft; Sarah Thaler, Anna Zorn, Isabell Aster, Dionysios Koliogiannis, Bernhard W. Renz, Markus Guba, and Philipp Groene reviewed and approved the final version of the manuscript.

## Conflicts of Interest

The authors declare no conflicts of interest.

## Data Availability

The data that support the findings of this study are available from the corresponding author upon reasonable request.
